# Comparative evaluation of soft tissue changes in Class I borderline patients treated with extraction and nonextraction modalities

**DOI:** 10.1590/2177-6709.21.4.050-059.oar

**Published:** 2016

**Authors:** Aniruddh Yashwant V., Ravi K., Edeinton Arumugam

**Affiliations:** 1 Senior lecturer, Department of Orthodontics and Dentofacial Orthopedics, Indira Gandhi Institute of Dental Sciences, MGMCRI campus, SBV University, Pillayarkuppam, Pondicherry, India.; 2 Professor and Head of Department, Department of Orthodontics and Dentofacial Orthopedics, SRM Dental College, Ramapuram, Chennai, India.; 3 Associate professor, Department of Orthodontics and Dentofacial Orthopedics, SRM Dental College, Ramapuram, Chennai, India.

**Keywords:** Angle Class I malocclusion, Borderline cases, Discriminant analysis, Soft tissue changes

## Abstract

**Objective:**

To compare soft tissue changes in Class I borderline cases treated with extraction and nonextraction modalities.

**Methods:**

A parent sample of 150 patients with Class I dental and skeletal malocclusion (89 patients treated with premolar extraction and 61 patients without extraction) was randomly selected and subjected to discriminant analysis which identified the borderline sample of 44 patients (22 extraction and 22 nonextraction patients). Pretreatment and post-treatment cephalograms of the borderline subsample were analyzed using 22 soft tissue parameters.

**Results:**

Upper and lower lips were more retracted and thickness of the upper lip increased more in the borderline extraction cases (*p* < 0.01). The nasolabial angle became more obtuse and the interlabial gap was reduced in the borderline extraction cases (*p* < 0.01). Lower lip, interlabial gap and nasolabial angle showed no changes in the borderline nonextraction cases.

**Conclusion:**

The soft tissue parameters which can be used as guideline in decision making to choose either extraction or nonextraction in Class I borderline cases are upper and lower lip protrusion in relation to the E-plane and Sn-Pg' line, lower lip protrusion in relation to the true vertical line (TVL), upper lip thickness, nasolabial angle and interlabial gap.

## RESUMO

**Objetivo:**

comparar as alterações sofridas nos tecidos moles em casos limítrofes de Classe I tratados com extrações e sem extrações.

**Métodos:**

uma amostra inicial de 150 pacientes com má oclusão esquelética e dentária de Classe I (89 pacientes tratados com extrações de pré-molares e 61 pacientes tratados sem extrações) foi aleatoriamente selecionada e submetida a uma análise discriminante, a qual permitiu selecionar uma amostra de 44 pacientes limítrofes (22 tratados com extrações e 22 tratados sem extrações). Telerradiografias obtidas antes e depois do tratamento dessa subamostra de pacientes limítrofes foram analisadas, utilizando-se 22 grandezas em tecidos moles.

**Resultados:**

nos casos limítrofes tratados com extrações, houve maior retração dos lábios superior e inferior e um maior aumento na espessura do lábio superior (*p* < 0,01); bem como o ângulo nasolabial tornou-se mais obtuso e o espaço interlabial sofreu redução (*p* < 0,01). Já nos casos limítrofes tratados sem extrações, o lábio inferior, o espaço interlabial e o ângulo nasolabial não apresentaram alterações significativas.

**Conclusão:**

as grandezas em tecidos moles que podem ajudar na tomada de decisão entre o tratamento com e sem extrações nos casos limítrofes de Classe I são: protrusão dos lábios superior e inferior em relação ao plano E e em relação à linha Sn-Pg', protrusão do lábio inferior em relação à linha vertical verdadeira (LVV), a espessura do lábio superior, o ângulo nasolabial e o espaço interlabial.

## INTRODUCTION

Orthodontics is the branch of Dentistry which mainly deals with malocclusion and dentofacial deformities and their correction for optimal function and esthetics. Orthodontic treatment should not focus only on occlusal relations, but also on facial esthetics, in particular profile esthetics, as they are the primary motive that encourages most patients to seek orthodontic treatment.^1^ In the present era, several treatment modalities emphasize soft tissue paradigm.^2^ ^,^ ^3^ Wuerpel E.H ^4^ discussed the changes in soft tissue that must be considered during orthodontic treatment, instead of moving teeth without anticipating soft tissue outcomes after treatment.

In treating a Class I malocclusion, there are two main approaches in comprehensive Orthodontics: extraction and nonextraction. Extractions are routinely used to correct dental crowding and protrusion of teeth and the overlying soft tissue. The nonextraction approach requires expansion of the arches, molar distalization or proximal stripping. The common demerits of extraction treatment were hypothesized to be "dished-in profiles," narrower dental arches, increased width of the buccal corridor; while those of nonextraction treatment were hypothesized to be poor stability and protrusive profile in borderline cases.^5^

There have been numerous studies about post-treatment soft tissue changes in Class II malocclusions, but the impact of facial esthetics in Class I cases has seldom been given importance.^6^ ^,^ ^7^ ^,^ ^8^ This study was undertaken to compare the soft tissue changes seen in extraction and nonextraction treatment modalities in Class I borderline malocclusions.

## MATERIAL AND METHODS

The treatment records of 150 patients with dental and skeletal Class I malocclusion were randomly selected from the record archive of patients treated over the past five years in the Department of Orthodontics and Dentofacial Orthopedics, SRM Dental College, Ramapuram, Chennai, India. Only patients whose treatment was finished with bilateral Class I canine and molar relationship were included in the study. Pretreatment and post-treatment cephalograms, which were taken from the same cephalostat with teeth occluding in centric occlusion and lips relaxed, were gathered. The study design was approved by the institutional Ethics Committee.

It is difficult to segregate borderline Class I malocclusions based only on specific parameters, especially when a large sample of patients is to be studied. Discriminant analysis is a multivariate statistical method wherein many parameters that influence treatment modality can be assessed. It can also help in identifying the predictors of treatment modality and also to identify borderline patients.^6^ ^,^ ^8^

Hence, in this study, a stepwise discriminant analysis was performed to segregate the borderline subsample of patients who could have been treated with either extraction or nonextraction treatment modalities. A total of 15 cephalometric variables, 4 model measurements, besides age and sex (demographic variables) were used for the discriminant analysis (Table 1). The values of the 21 variables were noted for all the 150 cases of the parent sample and data were subjected to discriminant analysis using Statistica software (StatSoft, Inc. USA). At each step of the discriminant analysis, all the 21 variables were reviewed and evaluated to determine which variable would contribute most to the discrimination between groups. That variable was then included in the discriminant model, and analysis was restarted. Thereby, the variables entered the discriminant function individually based on their discriminating power.

**Table 1:** Variables for discriminant analysis.

No	PARAMETERS	CHARACTERISTIC
1.	SNA	Maxillary position
2.	SNB	Mandibular position
3.	ANB	Maxillomandibular relationship
4.	FMA	Facial height/orientation of mandible
5.	U1-SN	Maxillary incisor protrusion
6.	U1-NA (linear)	Maxillary incisor protrusion
7.	U1-NA (angular)	Maxillary incisor inclination
8.	L1-NB (linear)	Mandibular incisor protrusion
9.	L1-NB (angular)	Mandibular incisor inclination
10.	Wits appraisal	Maxillomandibular relationship
11.	N-S-Ar	Mandibular position
12.	Z angle	Profile convexity
13.	L lip-E-plane	Lower lip protrusion
14.	L1-APog	Mandibular incisor position
15.	Jarabak ratio	Growth pattern/facial height
16.	Overbite	
17.	Overjet	
18.	Maxillary tooth material- arch discrepancy	
19.	Mandibular tooth material- arch discrepancy	
20.	Age	Demographic variable
21.	Sex	Demographic variable

Based on the data incorporated for the parent sample, only the variables that were significant were deemed eligible to be included in the discriminant analysis. From the inferential statistics, the discriminant function used three significant variables in descending order of importance, which were (*p* <0.01):

1. Maxillary tooth material - arch length discrepancy;
2. Mandibular tooth material - arch length discrepancy;
3. Mandibular incisor to NB (linear).

**Graph 1:**
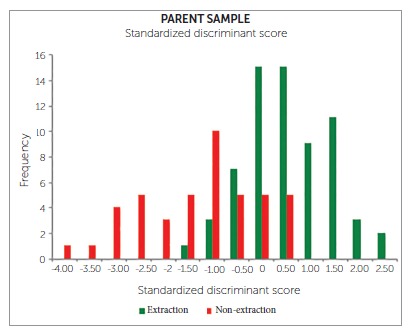
Standardized discriminant scores for parent sample.


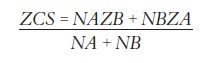



In which:

» Group A: Extraction.
» Group B: Nonextraction.
» ZCS: Critical cutting score between Group A and Group B.
» NA: Number of observations in Group A.
» NB: Number of observations in Group B.
» ZA: Centroid score for Group A.
» ZB: Centroid score for Group B.

The borderline subsample of patients was inferred to be those scores which were closest to the critical cutting score. Soft tissue landmarks were identified for soft tissue analysis of the 22 extraction and 22 nonextraction borderline cases, using the 22 parameters enlisted in Table 2 (Figs 1 to 13).

**Table 2:** Soft tissue analysis.

No	MEASUREMENT	DESCRIPTION
1.	G'-Sn-Pg' (Fig 1)	Angle of facial convexity
2.	Ls-E-plane (Fig 1)	Protrusion of the upper lip in relation to E-plane
3.	LL-E-plane (Fig 1)	Protrusion of the lower lip in relation to E-plane
4.	Ls-Sn-Pg' line (Fig 2)	Protrusion of the upper lip in relation to Sn-Pg' line
5.	LL-Sn-Pg' line (Fig 2)	Protrusion of the lower lip in relation to Sn-Pg' line
6.	Is-Stm [perpendicular to FH plane] (Fig 3)	Maxillary incisor exposure
7.	Is-Ls [on FH plane] (Fig 4)	Thickness of the upper lip
8.	Ii-LL [on FH plane] (Fig 4)	Thickness of the lower lip
9.	Max. Sulcus - Sn'-Ls (Fig 5)	Maxillary sulcus depth
10.	Mand. Sulcus - LL-Pg' (Fig 5)	Mandibular sulcus depth
11.	Nasolabial angle (Fig 6)	Formed by the intersection of labrale superius and columella at subnasale
12.	LLs - Me' (Fig 7)	Lower lip length
13.	Sn'- ULi (Fig 7)	Upper lip length
14.	ULi-LLs (Fig 7)	Interlabial gap
15.	G'-Sn' : Sn'-Me' (Fig 8)	Vertical height ratio
16.	ULi-Is (on TVL) (Fig 9)	Incisal exposure
17.	N' - Pn (perpendicular to True Vertical Line [TVL]) (Fig 10)	Projection of the nose
18.	N' - A' (perpendicular to TVL) (Fig 11)	Thickness of the upper lip
19.	N'- Ls (perpendicular to TVL) (Fig 11)	Protrusion of the upper lip
20.	N'- B' (perpendicular to TVL) (Fig 12)	Thickness of the lower lip
21.	N'-Li (perpendicular to TVL) (Fig 12)	Protrusion of the lower lip
22.	N'- Pg' (perpendicular to TVL) (Fig 13)	Soft tissue thickness at chin

**Figure 1:**
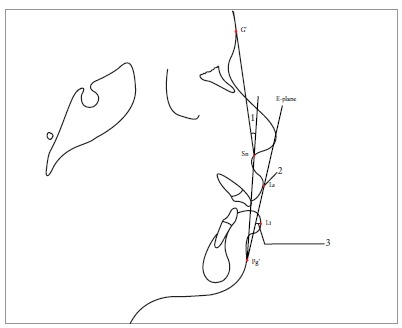
1 = Angle of facial convexity (G'-Sn-Pg'). 2 = Protrusion of upper lip (Ls to E-plane). 3 = Protrusion of lower lip (Li to E-plane).

**Figure 2:**
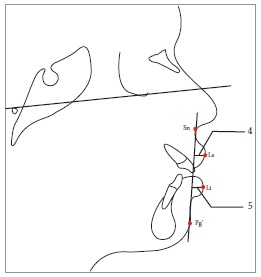
4 = Protrusion of upper lip (Ls-Sn-Pg' line). 5 = Protrusion of lower lip (Li-Sn-Pg' line).

**Figure 3:**
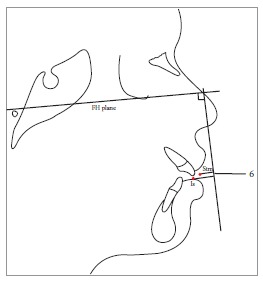
6 = Maxillary incisor exposure (Is-Stm)

**Figure 4:**
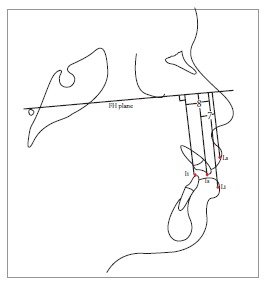
7 = Thickness of upper lip (Is-Ls).

**Figure 5:**
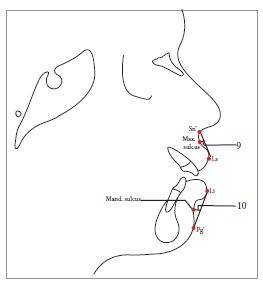
9 = Max. sulcus (Sn'-Ls). 10 = Mand. sulcus (Li-Pg').

**Figure 6:**
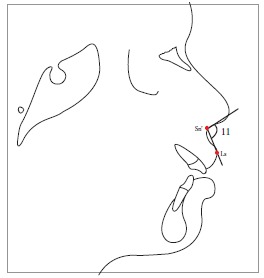
11 = Nasolabial angle.

**Figure 7:**
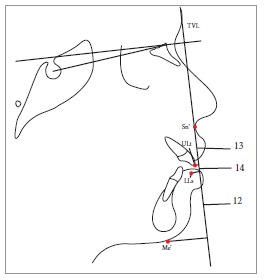
12 = Lower lip length (LLs - Me'). 13 = Upper lip length (Sn'-ULi). 14 = Interlabial gap (ULi-LLs).

**Figure 8:**
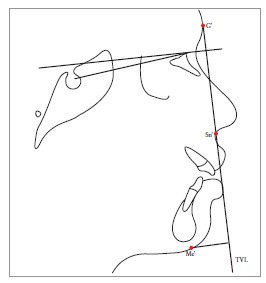
15 = Vertical height ratio (G'-Sn':Sn'-Me').

**Figure 9:**
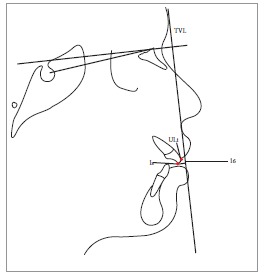
16 = Incisal exposure [ULi-Is (on TVL)].

**Figure 10:**
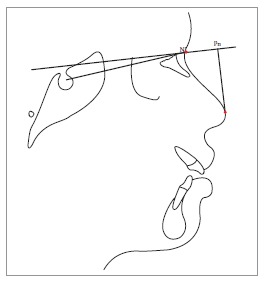
17 = N' - Pn (perpendicular to TVL).

**Figure 11:**
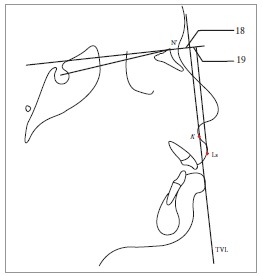
18 = N' - A' (perpendicular to TVL). 19 = N’- Ls (perpendicular to TVL).

**Figure 12:**
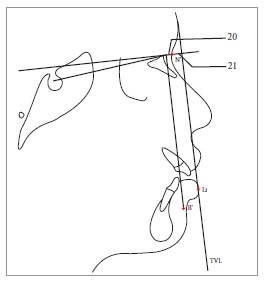
20 = N'-B' (perpendicular to TVL), 21 = N’-Li (perpendicular to TVL).

**Figure 13:**
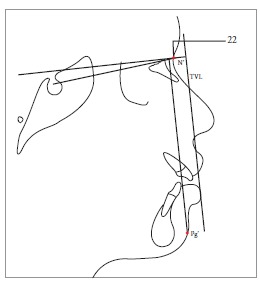
22 = N'-Pg' (perpendicular to TVL).

Ten random cephalometric radiographs were taken and assessed for the second time to test for the standard deviation of error in repeated measures for each soft tissue cephalometric measurement by means of Dahlberg's formula (√ (Σd)^2^/2N). 

Mean and standard deviation of the 22 soft tissue parameters were calculated for the extraction and nonextraction borderline samples before and after treatment. The mean and standard deviation for the differences that each treatment group experienced from pretreatment to post-treatment were also obtained. 

Independent sample t-tests were used to test the significance of differences between treatment change values of the two different treatment groups. The null hypothesis stating that no difference exists in the cephalometric variables in each treatment group before and after treatment was tested using paired t-tests (*p* < 0.05 was considered statistically significant). The standard deviation of error of the repeated measures for soft tissue cephalometric measurements was calculated by means of Dahlberg's formula.

## RESULTS

The descriptive and inferential statistics of all the 150 Class I cases using discriminant analysis are tabulated (Table 3). A total of 89 cases were treated by extraction of either first or second premolars and 61 cases by the nonextraction modality. Descriptive statistics of the parent sample of 150 cases showed that the sample consisted of patients with skeletal and dental Class I malocclusion. 

**Table 3:**
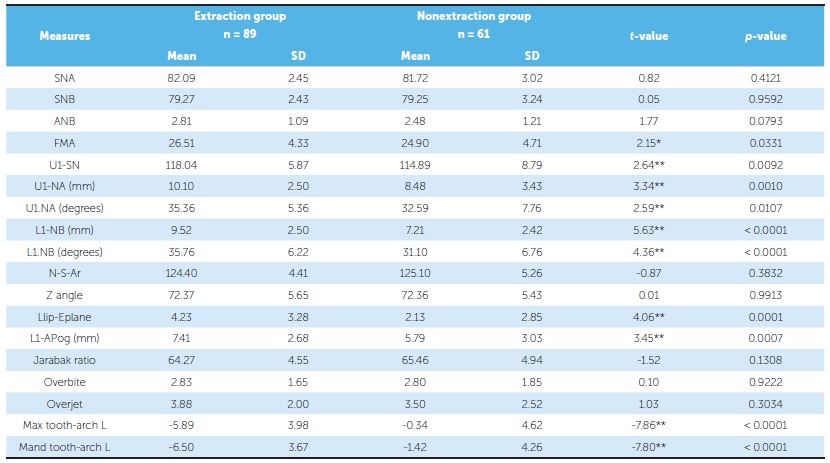
Descriptive statistics of the parent sample of 150 cases.

* p < 0.05 (Significant at 5%). ** p < 0.01 (Significant at 1%).

Out of ten significant parameters in the discriminant analysis, maxillary tooth material-arch length discrepancy (Max tooth-arch length) is the most important in differentiating extraction and nonextraction groups, followed by mandibular tooth material-arch length discrepancy (Mand tooth-arch length) and linear relationship of mandibular incisor to NB [L1-NB(L)], as shown in Table 4. 

**Table 4:**
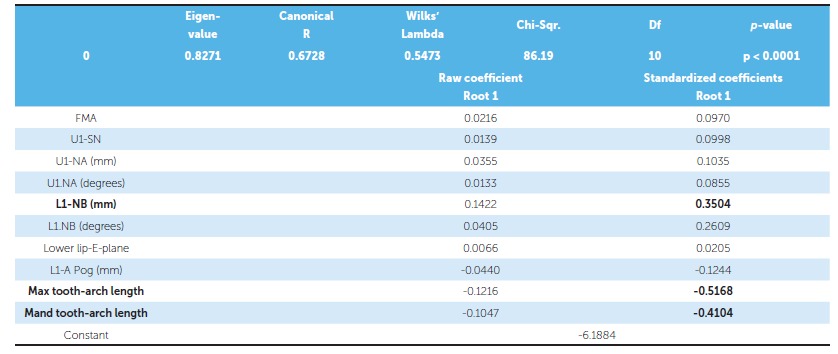
Discriminant analysis: significance of the function differentiating extraction and nonextraction cases.

Comparative statistics of the borderline extraction sample and borderline nonextraction sample is listed in Tables 5 and 6, respectively. Upper lip thickness increased significantly from 12.09 mm at treatment onset to 14.02 mm at the end of treatment in the borderline nonextraction sample. The other parameters did not show statistically significant changes.

**Table 5:**
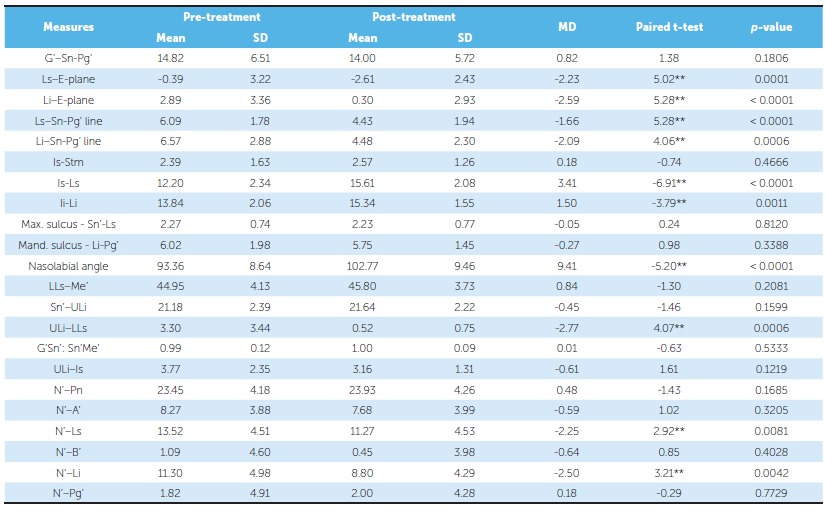
Borderline extraction sample: descriptive and inferential statistics of soft tissue analysis results.

**Table 6:**
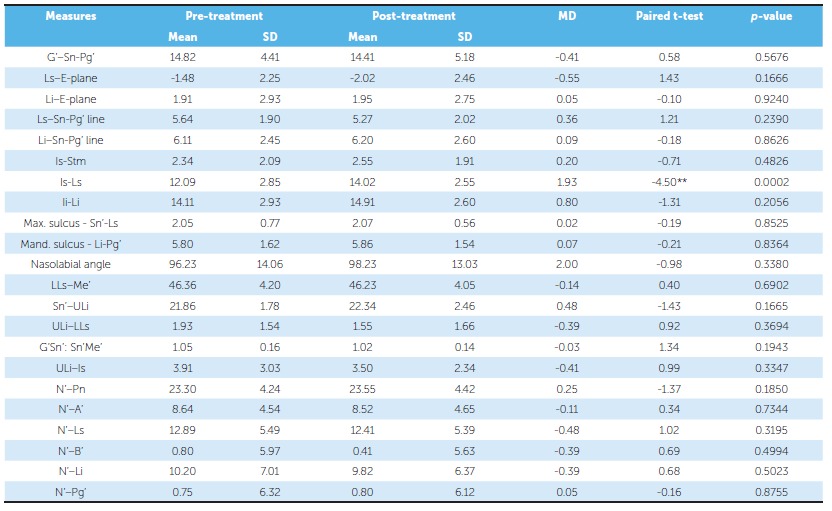
Borderline nonextraction sample: descriptive and inferential statistics of soft tissue analysis results.

Comparative statistics of mean differences between extraction and nonextraction borderline samples are listed in Table 7. In relation to the E-plane, the upper lip was retracted by 2.23 mm in the extraction and by 0.55 mm in the nonextraction group; whereas the lower lip was retracted by 2.59 mm in the extraction and by 0.05 mm in the nonextraction group. The mean soft tissue change values for the upper lip in relation to the Sn-Pg' line were -1.66 mm for the extraction and -0.36 mm for the nonextraction group; whereas for the lower lip in relation to the Sn-Pg' line, the mean change values were -2.09 mm for the extraction and 0.09 mm for the nonextraction group. The mean soft tissue change values for the lower lip in relation to true vertical line (TVL) were -2.50 mm for the extraction and -0.39 mm for the nonextraction group. Upper lip thickness increased by 3.41 mm in the extraction and 1.93 mm in the nonextraction group. The increases in nasolabial angle were 9.410° in the extraction group and 20° in the nonextraction group. Interlabial gap decreased by 2.77 mm in the extraction group and by 0.39 mm in the nonextraction group.

**Table 7:**
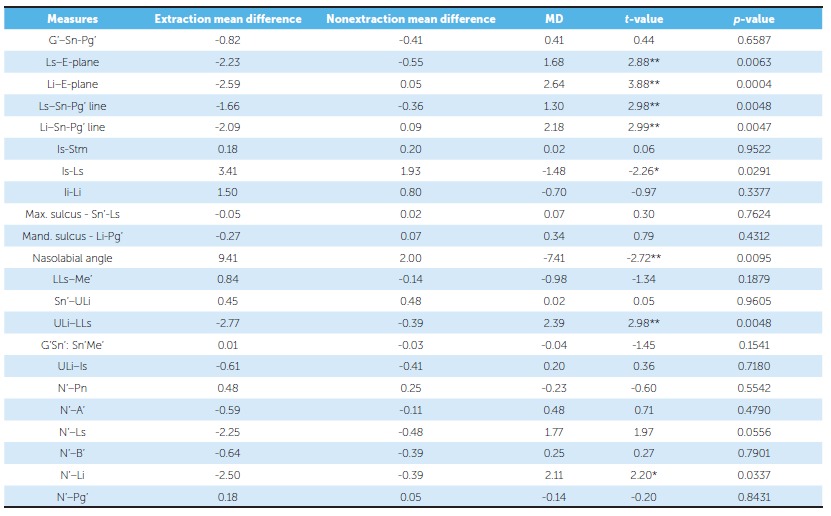
Descriptive and inferential statistics of mean value differences: extraction *versus* nonextraction.

*p < 0.05 (Significant at 5%). **p < 0.01 (Significant at 1%).

The values of standard deviation of error of the repeated measures for each of the soft tissue cephalometric measurement by means of Dahlberg's formula are listed in Table 8. These values were found to be comparable to those reported in the literature.^6^ ^,^ ^10^ ^,^ ^11^

**Table 8:** Standard deviation of error for repeated measures.

Parameters	Standard deviation of error
G'-Sn-Pg'	0.7416
Ls-E-plane	0.1936
Li-E-plane	0.3162
Ls-Sn-Pg'	0.5916
Li-Sn-Pg'	0.2958
Is-Stm	0.3708
Is-Ls	1.0124
Ii-Li	0.5123
Max. sulcus (Sn'-Ls)	0.4031
Mand. sulcus (Li-Pg')	0.3873
Nasolabial angle	3.6125
Lower Lip length	0.5701
Upper lip length	0.6021
Interlabial gap	0.1936
G'Sn':Sn'Me'	0.0647
Uli-Is	0.3708
TVL N'-Pn	0.3708
TVL N'-A'	0.4472
TVL N'-Ls	0.9421
TVL N'-B'	0.6124
TVL N'-Li	0.3354
TVL N'-Pg'	0.3354

## DISCUSSION 

There is probably no other aspect of orthodontic treatment that has caused as much controversy as the decision of whether to extract or not permanent teeth. Just like a pendulum, the popularity of premolar extractions has swung between the option of nonextraction at any cost and extraction treatment to achieve arbitrary cephalometric norms.

Borderline cases are those cases which are equally susceptible to both extraction and nonextraction treatment modalities. The aim of this study was to compare soft tissue changes in Class I borderline cases treated with extraction and nonextraction modalities and to identify those parameters which can act as guidelines to differentiate between these two treatment modalities in Class I borderline cases.

Considering the changes in the upper lip in relation to E-plane, the borderline extraction sample showed -2.23-mm retraction while the borderline nonextraction sample showed -0.55-mm retraction. Drobocky et al.^12^ and Bravo^13^, in their studies, reported -3.4 mm of upper lip retraction with extraction of maxillary first premolars. Kocadereli^14^, in his study, showed that upper lip was retracted by -1.64 mm. Upper lip retraction in relation to the true vertical line was found to be -2.25 mm for the extraction group and -0.48 mm for the nonextraction group. In relation to the Sn-Pg' line, upper lip protrusion was reduced by -1.66 mm in the extraction group and was insignificant in the nonextraction group. Drobocky et al.^12^ and Bravo^13^ reported upper lip retraction in relation to Sn-Pg' line values to be of -2.12 mm and -2.4 mm, respectively. The insignificant reduction in lip protrusion in the nonextraction group is similar to the values seen in the studies by Kocadareli^14^ and Konstantonis^15^.

Upper lip thickness was increased by 3.41 mm in the extraction group and by 1.93 mm in the nonextraction group. These values are comparable to the study results of Talass et al^16^ who reported an increase of upper lip thickness of 3.7 mm in the extraction group.

The nasolabial angle showed an increase of 9.41^°^ in the extraction borderline group. Bravo reported an increase of 3.7^°^ in nasolabial angle with the extraction of first premolars.^13^ Ramos et al^17^ reported an increase of 4^°^ in their study which involved extraction of maxillary first premolars for treatment of Class II, Division 1 cases. The increase in the nasolabial angle was statistically insignificant in the nonextraction group. Contrary to the results obtained in our study, Waldman^18^ reported that there was only a slight correlation (r = 0.42) between retraction of anterior teeth and change in the nasolabial angle.

The changes in lower lip showed significant difference between treatment groups. In relation to the E-plane, the lower lip was retracted by -2.59 mm in the extraction group. Drobocky et al.^12^ reported a similar value of lower lip retraction with extraction of first premolars (-3.22 mm). In the nonextraction group, lower lip in relation to E-plane showed no change. Konstantonis^15^, in his study, showed that the lower lip was brought forward by 0.67 mm. In contrast to these findings, Battagel, Finnoy et al and Xu et al reported lower lip retraction with values of -1.44 mm, -2.2 mm and -0.4 mm, respectively.^10^ ^,^ ^19^ ^,^ ^20^ With respect to the Sn-Pg' line, the lower lip showed -2.09-mm retraction in the extraction group and no change in the nonextraction group. The findings by Konstantonis^15^ showed -2.55-mm retraction in the extraction group and 1.01-mm lower lip protraction. Young and Smith^11^ found -0.58-mm lower lip retraction. The mean values of lower lip response to treatment vary between this study and the other studies discussed above. This can be due to factors such as variation in position of the maxillary incisor post-treatment, weak correlation between mandibular incisors retraction and lower lip position, as well as weaker correlation and ratio between lower lip change and underlying hard tissue change due to treatment. In relation to the true vertical line (TVL) the lower lip showed 3.21-mm retraction in the extraction group. The change in the nonextraction group was insignificant. These values were comparable to the values inferred from lower lip changes in relation to the Sn-Pg' line and E-plane. Hence, the relationship between soft tissue landmarks and the true vertical line (TVL) shows that it can be used as an adjunct parameter for assessing soft tissue changes with treatment.

The interlabial gap was found to reduce by 2.77 mm in the extraction group. This parameter did not show any significant change with nonextraction treatment. Jacobs^21^, in his study, reported that the decrease in interlabial gap can be predicted by retraction and intrusion of maxillary incisors. The change in interlabial gap was found only in the extraction group, probably because of significant lower lip retraction (-2.59 mm in relation to E-plane). This inference can be confirmed with the results of a study by Yogosawa^22^, which showed that to close interlabial gap, movement of lower lip must be four times the movement of upper lip. Contrary to these results, Janson et al^23^ reported that nonextraction patients had greater interlabial gap reduction (2.7 mm) than observed in extraction patients (1.3 mm) in the long-term post-treatment period. 

There exists a difference in treatment changes between this study and those carried out by other authors discussed herein. Soft tissue changes due to extraction or nonextraction treatment depend on the characteristics of the patients studied, sample size, the prescription used, anchorage considerations and treatment mechanics. Many of the studies discussed above have shown soft tissue changes associated with Class II malocclusions.^13^ ^,^ ^16^ ^-^ ^19^ Moreover, treatment mechanics and anchorage considerations were not specified in many of those studies. This influences the amount of incisor retraction which, in turn, influences soft tissue changes. 

 In this study, all patients were treated by MBT prescription in 0.022-in slot with appropriate anchorage preparation. Few of the studies discussed have used Tweed's technique. It has been shown that patients treated with Tweed's technique have shown greater lip retraction.^12^ These may be the reasons why the values of soft tissue changes of this study do not coincide with values observed in other studies.

## CONCLUSION

From the results obtained in this study, it can be concluded that upper and lower lips were retracted more significantly, while upper lip thickness increased more significantly in the borderline extraction cases. The nasolabial angle became more obtuse and the interlabial gap was reduced in the borderline extraction cases. The other parameters, such as maxillary incisor exposure, upper and lower lip lengths, vertical height ratio and soft tissue changes at the chin, were found to be statistically insignificant in both extraction and nonextraction treatment groups. 

The parameters which differentiate between extraction and nonextraction treatment modalities in Class I borderline cases are upper and lower lip protrusion in relation to E-plane and the Sn-Pg' line, lower lip protrusion in relation to the true vertical line (TVL), upper lip thickness, nasolabial angle and interlabial gap. These parameters can be used as guidelines in decision making to choose either extraction or nonextraction in Class I borderline cases.
